# Supplementary material for: Polyketide synthase gene clusters in the ericoid mycorrhizal fungus Oidiodendron maius: genome-wide prediction, expression profiling, and functional characterization of knockout mutants
Source: Front Plant Sci. 2026 Sep 16;17:1924430. doi: 10.3389/fpls.2026.1924430 (PMC13623903; doi:10.3389/fpls.2026.1924430)
Supplement: Supplementary file 1 [file SupplementaryFile1.zip › Supplementary Materials/Supplementary Folder 1.zip!/Oidma1_AssemblyScaffolds_Repeatmasked/knownclusterblast/cluster56/jgi.p_Oidma1_138909_mibig_hits.html]

| MIBiG Protein | Description | MIBiG Cluster | MiBiG Product | % ID | % Coverage | BLAST Score | E-value |
| --- | --- | --- | --- | --- | --- | --- | --- |
| AGA37279.1 | P450\_monooxygenase | BGC0000819 | Paraherquamide\_biosynthetic\_gene\_cluster | 31 | 103.389830508 | 276 | 7.3e-74 |
| AIA58897.1 | putative\_cytochrome\_P450 | BGC0001141 | Brefeldin\_biosynthetic\_gene\_cluster | 33 | 88.1355932203 | 272 | 8.1e-73 |
| OAQ83757.1 | cytochrome\_P450 | BGC0001358 | Leucinostatins\_biosynthetic\_gene\_cluster | 30 | 103.389830508 | 242 | 1.5e-63 |
| BAO10618.1 | cytochrome\_P450\_monooxygenase | BGC0001262 | AK-toxin\_biosynthetic\_gene\_cluster | 34 | 34.8399246704 | 102 | 1.1e-21 |
| AAK53577.1 | trichodiene\_oxygenase | BGC0000930 | Trichothecene\_biosynthetic\_gene\_cluster | 23 | 87.0056497175 | 96 | 1e-19 |
| EFY94491.1 | cytochrome\_P450,\_putative | BGC0000337 | Destruxins\_biosynthetic\_gene\_cluster | 25 | 70.9981167608 | 84 | 3.1e-16 |
| EHA28236.1 | monooxygenase | BGC0001143 | Azanigerone\_biosynthetic\_gene\_cluster | 31 | 34.0866290019 | 78 | 2.2e-14 |
| chr3.CM0292.110.r2.m |  | BGC0001317 | Lupeol\_biosynthetic\_gene\_cluster. | 33 | 32.2033898305 | 67 | 4e-11 |
| ACH72913.2 | AflN | BGC0000011 | Aflatoxin/sterigmatocystin\_biosynthetic\_gene\_cluster | 31 | 34.0866290019 | 67 | 6.8e-11 |
| BAC20565.1 | cytochrome\_P450 | BGC0000039 | Compactin\_biosynthetic\_gene\_cluster | 30 | 25.8003766478 | 50 | 5e-06 |
